# Correction to: The Pathfinder plasmid toolkit for genetically engineering newly isolated bacteria enables the study of *Drosophila*-colonizing *Orbaceae*

**DOI:** 10.1038/s43705-023-00329-2

**Published:** 2023-11-16

**Authors:** Katherine M. Elston, Laila E. Phillips, Sean P. Leonard, Eleanor Young, Jo-anne C. Holley, Tasneem Ahsanullah, Braydin McReynolds, Nancy A. Moran, Jeffrey E. Barrick

**Affiliations:** 1 Department of Molecular Biosciences, The University of Texas at Austin, Austin, TX 78712 USA; 2 Department of Integrative Biology, The University of Texas at Austin, Austin, TX 78712 USA; 3 Freshman Research Initiative, The University of Texas at Austin, Austin, TX 78712 USA

**Keywords:** Microbiome, Bacterial genetics

Correction to: *ISME Communications* 10.1038/s43705-023-00255-3; published online 24 May 2023

When using the Pathfinder plasmids in further studies, we discovered that the wrong plasmid had been used in all tests of pSL9 reported in our article. A plasmid with a pUC origin of replication was accidentally used in place of the intended plasmid with a broad-host-range pBBR1 origin of replication. This error affects results reported in Fig. 1B and Fig. 2B. To address this error, we constructed a corrected version of the pSL9 plasmid and repeated these assays.

We found that the phenotypes of colonies of *E. coli* MFD*pir* cells transformed with the corrected pSL9 plasmid closely resemble the images shown in Fig. 1B. These colonies are still easily distinguishable from those formed by cells transformed with the other Pathfinder plasmids by their appearance under white and blue light illumination and their fluorescence at different excitation and emission wavelengths. The overall expression level of the E2-Crimson reporter gene from the updated plasmid is similar to what was observed for the pUC plasmid.

Next, we repeated a representative subset of the conjugation assays reported in Fig. 2B. In these tests, we conjugated from just one donor *E. coli* MFD*pir* strain transformed with the pSL9 plasmid rather than from a pool of donors containing multiple Pathfinder plasmids. We successfully obtained transconjugants of the *E. coli* DH5α control, as was the case in the original results with the incorrect pUC version of the plasmid. Now, however, we also obtained transconjugants of the lpD01 *Orbaceae* strain when using the corrected pBBR1 version of the pSL9 plasmid. It is likely that this plasmid would also replicate in the other *Orbaceae* strains if they were retested. To further validate the functionality of the new plasmid, we also verified that the updated pSL9 plasmid could be conjugated into the bee gut bacterium *Snodgrassella alvi* wkB2, whereas the pUC version did not yield any transconjugants with this host.

We have replaced the original pSL9 (pBTK1009) plasmid that was deposited in Addgene to accompany this article with the corrected pBBR1 version (Addgene: #191004).

We thank A H M Zuberi Ashraf and Kadena Cope for testing the corrected pSL9 plasmid.

